# Suicide in veterinary medicine: A literature review

**DOI:** 10.14202/vetworld.2023.1266-1276

**Published:** 2023-06-08

**Authors:** Carina Rodrigues da Silva, Ana Amélia Domingues Gomes, Thaís Rabelo dos Santos-Doni, Alexandre Coutinho Antonelli, Rafael Felipe da Costa Vieira, Alexandre Redson Soares da Silva

**Affiliations:** 1 Department of Veterinary Medicine, Universidade Federal do Vale do São Francisco, Campus of Agricultural Sciences, Petrolina, Pernambuco, Brazil; 2 College of Veterinary Medicine, Department of Veterinary Preventive Medicine, Ohio State University, Columbus, OH, USA; 3 Department of Veterinary Medicine, Universidade Federal do Vale do Jequitinhonha e Mucuri, Unaí, Minas Gerais, Brazil; 4 Department of Public Health Sciences, College of Health, and Human Services, University of North Carolina, Charlotte, North Carolina, United States of America; 5 Center for Computational Intelligence to Predict Health and Environmental Risks, University of North Carolina, Charlotte, North Carolina, United States of America

**Keywords:** burnout, depression, mental health, occupational stress, veterinarians

## Abstract

Veterinarians are commonly exposed to occupational stressors, including excessive workload and financial constraints. These stressors can lead to psychological distress, which typically results in mental health disorders such as depression, anxiety, and burnout and can even culminate in suicide attempts or suicide deaths. Risk factors associated with poor mental health and high rates of suicide in veterinary practitioners include continuous exposure to challenging scenarios, such as interpersonal conflicts, performing euthanasia, and easy access to lethal means of suicide, such as opioids and anesthetics. The previous studies highlight the urgent need for a better understanding of predisposing factors, mental health-related improvements in the professional environment, and the subsequent establishment of primary mental health-related care policies. Effective ways to promote mental health and prevent suicide may include social support, resilience, developing coping skills, promoting a healthy work environment, and discouraging perfectionist behaviors. This review aimed to summarize findings in studies that have investigated mental health and suicide in veterinarians and veterinary students and highlight measures that could be implemented as options for mental health promotion and suicide prevention.

## Introduction

Veterinarians are at elevated risk of developing mental health disorders and dying by suicide [1, 2], likely influenced by high levels of stress and poor mental health [2, 3]. However, as incisively stated by Moutier and Mortali [4], “we can no longer accept as status quo that suicide rates are higher among veterinary professionals compared with the general population.” There is considerable evidence that veterinarian practitioners and veterinary students experience high depression, anxiety, and burnout rates [1, 5–7]. Occupational stressors reported in published studies include long working hours, client expectations, unexpected patient outcomes, communication of bad news to pet owners, high workloads, rising veterinary care costs, professional isolation, and occupational imbalance [2, 8, 9]. Moreover, veterinarians often exposed to psychological distress from challenging scenarios, such as interpersonal conflicts and performing euthanasia, are even more predisposed to poor mental health [3].

It is paramount that we endeavor to mitigate such outcomes by identifying risk factors for suicidal behavior, including ideation, attempt, and death [10].

However, there is still little information available on preventive measures for suicide among veterinary practitioners, specifically in veterinary settings. Therefore, this manuscript aimed to summarize findings on the risk factors for suicide in veterinarians and highlight measures that could be effective options for mental health promotion and suicide prevention.

## Suicide Rates in Veterinary Medicine

According to the World Health Organization (WHO), a person dies by suicide every 40 s [11]. This is particularly of concern for the veterinary medicine field since the previous studies have shown that suicide rates for these professionals are higher than those within the general population [12–15]. Table-1 summarizes a few studies that addressed suicide among veterinarians, veterinary professionals, and students, collected by non-systematic review in updated scientific databases [2, 12, 16–18].

**Table-1:** Comparative table of studies addressing suicide in veterinarians, veterinary professionals, and students, collected by non-systematic review in updated scientific databases.

Reference	Sample Size (study method), population (location)	Analyzed conditions	Key findings – rate/risk frequency (%)		

			VP	VS	GP
[31]	913 (survey) – Veterinary students (Germany).	Depression	-	45.90%	3.20%
		Suicide ideation	-	19.90%	4.50%
		Suicide risk	-	24.00%	6.60%
[12]	3,118 (survey) – Veterinarians (Germany).	Depression	27.78%	-	3.99%
		Suicide ideation	19.20%	-	5.70%
		Suicide risk	32.11%	-	6.62%
[98]	3,540 (survey) – Veterinarians (US).	Suicide ideation	24.90%	-	N/A
		Suicide attempts	1.60%	-	5.10%
[99]	10,254 (survey) – Veterinarians (US).	Psychological distress	6.80% (M) 10.90% (F)	-	3.50% (M) 4.40% (F)
		Depression	24.50% (M) 36.70% (F)	-	15.10% (M) 22.90% (F)
		Suicide ideation	14.40% (M) 19.10% (F)	-	5.10% (M) 7.10% (F)
		Suicide attempts	1.10% (M) 1.40% (F)	-	1.60% (M) 3.00 (F)
[2]	11,627 (survey) – Veterinarians (US).	Depression	31% (1:6 psychological distress)	-	-
		Suicide ideation	17% (1:11)	-	-
		Suicide attempts	1%	-	-

GP=General population, M=Males, F=Females, VP=Veterinary professionals, VS=Veterinary students

For instance, a retrospective study (1979–2015) compared causes of death using proportional mortality ratios of North American veterinarians with the general US population [19] using life insurance data provided by the American Veterinary Medical Association (AVMA) and data from the Central Bank of Death Records of the United States of America (US). The study showed that 398/11,620 (3.42%) veterinarians died from suicide, with 326 (82%) of the decedents being male and 298 (75%) dying at ≤65 years of age (which are considered “working ages”). The proportional mortality ratios for the suicide of veterinarians were significantly higher than that of the North American general population. Most suicide cases were among men, with a proportional mortality ratio of 1.8; however, women were 3.4 and 5.0 times more likely to die by suicide than the general population when evaluated based on the field of work, in clinical and non-clinical roles, respectively [19].

Another study by Witte *et al*. [14] analyzed death records of 202 veterinary professionals and veterinary students with a cause of death characterized as “suicide” or “undetermined intent” from 2003 through 2014. This study used standardized mortality ratios and considered circumstances surrounding the deaths, which provided more detailed information on suicide methods for this population. These circumstances included psychological/psychiatric treatment and occupational and relationship problems. From the 202 suicide records, standardized mortality ratios for veterinarians were 1.6 and 2.4 times higher for men and women, respectively, compared to the general population. Surprisingly, suicide rates for veterinary technicians or technologists were also significantly higher than that of the general population: 5.0 times higher for men and 2.3 times higher for women [14].

## Suicide Risk Factors

### Mental health disorders

Suicidal ideation can be triggered by worsening mental illness and/or extenuating problems in an individual’s personal or professional life; it is a dangerous moment when the individual gives up on their life [20]. Suicide is associated with mental health disorders such as depression, substance abuse disorders, and psychosis, and also with anxiety and other disorders related to personality, eating, and trauma [21–23].

Depression or major depressive disorder is the most common mental health disorder associated with suicide [24]. A state of decreased interest or pleasure in almost every daily activity affects sleep quality, leading to fatigue or energy loss, excessive weight gain or loss, reduction or gain in appetite, agitation, or psychomotor delay [25]. It also interferes with one’s mental state, causing feelings of worthlessness, indecisiveness, excessive or inappropriate guilt, and reduced ability to think or concentrate, sometimes leading to suicidal intent with recurring thoughts of death [5, 26]. In contrast, anxiety disorder is characterized by primary symptoms that are not commonly associated with other mental health conditions. Some examples of anxiety disorders are panic, obsessive-compulsive disorder, separation, social anxiety, and generalized anxiety disorders [27]. Their emotional, somatic, and behavioral manifestations include muscle tension, palpitations, difficulties in perception and learning, and high emotionality [28]. Although less severe than depression, anxiety disorders can also be related to suicide attempts [29]. Nepon *et al*. [29] evaluated more than 34,000 adults in the US between 2004 and 2005 through the National Epidemiologic Survey on Alcohol and Related Conditions-II representative longitudinal epidemiologic face-to-face survey of the civilian, non-institutionalized adult population of the US aged 18 years and older. They observed that the presence of an anxiety disorder, especially post-traumatic stress disorder and panic disorder, was significantly and independently associated with suicide attempts in over 70% of the analyzed individuals of the study [29].

Notably, there is a significant prevalence of depression and anxiety disorders among veterinary professionals and undergraduate students [16, 30–33]. A previous study involving 264 veterinary students in a North American university using the instrument “Patient Health Questionnaire-4” [34] detected high levels of stress, anxiety, and depression among participants. Approximately 23% of students were diagnosed as having depression, according to the tool’s measurements – a higher rate compared to the general population (16.6%) and medical students (14.3%) [6]. It was also observed that students with low grades were more susceptible to anxiety disorders [6].

In another study, 11,627 North American veterinarians were assessed using an online questionnaire to investigate risk factors for suicide, attitudes related to mental illness, and occupational stressors [2]. Results showed that one out of every 11 early-career veterinarians (<5 years of experience) had severe psychological distress, a state of emotional turbulence manifested by depressive and anxiety symptoms. Since graduating from veterinary school, 3655 (31%) respondents experienced depressive episodes, 1952 (17%) experienced suicidal ideation, and 157 (1%) attempted suicide [2].

Finally, although not directly related to suicide [35], burnout can also be a potential cause of mental illness and worsen depressive symptoms. Burnout is defined as a syndrome characterized by continuous stress, physical and mental exhaustion that develops slowly and leads to energy exhaustion due to specific requests, depersonalization, and lack of professional achievement [36]. In a 2016–2018 AMVA Census study involving 5002 full-time veterinarians, 50.2% showed signs of burnout [37]. Similarly, a study with Australian veterinarians revealed that recently graduated professionals had higher levels of psychological distress when compared to veterinarians that had graduated earlier; thus, early-career graduates could experience burnout more intensely [38].

### Personality traits

When considering vulnerability, Lewis and Cardwell [39] and Crane *et al*. [9], found that extreme perfectionism in morally stressful situations makes individuals more vulnerable to moral distress and reduces well-being [9, 39]. For example, when a veterinarian considers only one therapeutic approach appropriate, they may consider other options unacceptable. Whenever opinions between other professionals or the owner differ from the expected, a stress reaction may be triggered. A less perfectionist clinician may be able to think through situations better, stay calm, and find a way to work with the animal owner under the circumstances they are facing. It is important to emphasize that professional perfection is impractical and impossible to achieve. Still, there should be a distinction between the desire for morally consistent results and achievable moral standards [9, 39].

The “Big Five” personality traits theory, proposed by McCrae and Costa [40], comprises five main traits: extraversion (or extroversion), agreeableness, openness, conscientiousness, and neuroticism [40]. Among veterinary graduates, neuroticism (the tendency to feel continuous negative emotions) stands out as the personality trait that most significantly predicts occupational stress [40]. High levels of neuroticism, in association with low levels of conscientiousness (being efficient, organized, careful, or diligent), have strong relationships with stress, depression, and dysfunctional coping (lack of an individual’s efforts to regulate stressful situations) [41]. An environment that intensifies certain susceptible personality types and the uncertain etiology of all the professional issues regarding veterinary well-being may lead to recruiting those at an elevated risk of developing mental health disorders considered “risky phenotypes” [42].

### Occupational stressors

Despite the importance of mental health disorders in suicide ideation and attempts, other variables can influence the association between occupation and suicide, including work-related stressors, low job security, low pay, socioeconomic status, economic and work climate, and gender [43]. Likewise, high workload and long hours of work, personal and financial worries (e.g., student debt), rising veterinary care costs, unrealistic client expectations, communication of bad news to clients, unexpected patient outcomes, lack of senior support, professional isolation, and poor work-life balance can all be negative contributors (Table-2) [2, 17, 44, 45]. Becoming a veterinarian demands a complex interaction between personality traits, technical knowledge, and adaptation to the work environment, and the presence of high levels of stress and a stressful work climate could at least partially explain the higher levels of depression among veterinarians [46, 47].

**Table-2:** Comparative table of studies addressing occupational risk factors to suicide and mental health issues in veterinarians, collected by non-systematic review in updated scientific databases.

Reference and country	Sample	Total sample size (response rate), design	Evaluation tool	Exposure	Outcome	Main results
[45], UK	Veterinary surgeons	1,796 (56%), cross-sectional survey	HSE MS; HADS-A/D; WEMWBS	- Number of hours worked - Client expectations - Making professional mistakes - Administrative and clerical tasks - Client complaints or litigation - Unexpected clinical outcomes - Out-of-hours on-call duties	Work-related stress; anxiety; depression	43%*, 2.24** (n = 1,756) 28%*,1.83** (n = 1,755) 40%*, 2.22** (n = 1,747) 38%*, 2.12** (n = 1,740) 43%*, 2.19** (n = 1,583) 40%*, 1.98** (n = 1,578) 38%*, 1.93** (n = 1,512)
[44], Norway	Veterinarians	2,596 (75%), cross-sectional survey	PQ; BCI; SCL-5; CJSQ	- Emotional demands - Work-life balance - Fear of complaints/criticism	Serious suicidal thoughts	OR = 1.12, 95% CI (1.08–1.16) OR = 1.13, 95% CI (1.09–1.17) OR = 1.18, 95% CI (1.11–1.25)
[2], USA	Veterinarians	11,627 (NA), cross-sectional survey	K-6 PDS	- Demands of practice (work overload, long work hours) - Practice management responsibilities - Making professional mistakes - Client complaints - Dealing with personal, staff, or client grief - Unrealistic client expectations - Animal deaths (from illness or euthanasia) - Ethical challenges - Fear of malpractice litigation - Educational debt - Poor social support - Unclear management and work role - Lack of participation in decision-making	- Serious psychological distress - Depressive episodes - Suicide ideation - Suicide attempt	9% (n = 1,077) 31% (n = 3,655) 17% (n = 1,952) 1% (n = 157)
[17], USA	Veterinarians	3,540 (17.7%), cross-sectional survey	K-6 PDS	- Workload (>45 h/week) - Working on evening hours - Working more hours than desired - Working fewer hours than desired - Student debts	Serious psychological distress.	6.9% (n = 1,364) 9.4% (n = 1,169) 8.4% (n = 1,581) 6.4% (n = 156) 5.3% (n = 3,539)

HSE MS=Health and Safety Executive Management Standards Indicator tool, HADS-A/D=Hospital Anxiety and Depression Scale – anxiety/depression, WEMWBS=Warwick-Edinburgh Mental Well-being Scale, PQ=Paykel’s Questionnaire, BCI=Basic Character Inventory, SCL-5=Symptom Check-list-5, CJSQ=Cooper’s Job Stress Questionnaire, K-6 PDS=Kessler-6 psychological distress scale, NA=Not applicable.

* Percentage of respondents reporting that these stressors contribute “quite a lot” or “very much” to their stress,

** Mean scores in the HADS-A/D questionnaire

According to Skipper and Williams [48], the lack of awareness of their mental vulnerability can also increase the risk of suicide among veterinarians [48]. In this case, a better understanding of risk factors for mental health disorders, especially depression, is critical because both are risk factors for suicide, and suicidal ideation is a crucial precursor to death by suicide [22, 49].

Occupational stress occurs when an individual perceives the work environment as a threat, whether for personal or professional reasons, and demands are more significant than the ability to accomplish work tasks [50]. When a stress stimulus becomes chronic or intense, technically termed the exhaustion phase, severe physical and mental health consequences can occur [51–53]. Over the past decade, many studies have shown that occupational stressors can be related to the occurrence of suicide [54]. Job stressors that were associated with an elevated risk of suicide ideation and behaviors included exposure to lower supervisory and collegial support; and low job control, which is characterized as a limited ability to learn new things or develop skills and a lack of decision-making ability [54]. Work-life balance has also been shown to be a common source of job-related stress. Sirgy and Lee [55] define the work-life balance as “a high level of engagement in work-life as well as non-work-life with minimal conflict between social roles in work and non-work-life” [55]. It is noted that the poor balance between professional and personal life can directly influence outcomes in job satisfaction, as well as in physical health (smoking, cardiovascular and musculoskeletal disorders, stress, and fatigue) [56], and mental health, such as depressive symptoms and anxiety disorders, especially in women [57]. Therefore, Kersebohm *et al*. [58] suggest that combining a better balance between personal and professional life and reducing working hours can improve the satisfaction of veterinarians [58].

Veterinary practitioners can also face moral and ethical conflicts, leading to frustration and emotional distress [59, 60]. Moral distress is the experience of psychological distress resulting from being involved or failing to prevent decisions or behaviors that violate or may violate, personal, moral, or ethical beliefs [61]. Moral distress is considered an occupational stressor and a source of psychological pressure, and it is common in various professions (e.g., the human medical field) [62]. Individuals subjected to moral distress cannot accomplish things that they normally could perform easily, contributing to judgment mistakes, personal flaws, and character weaknesses in the work environment [60, 63]. Moral challenges do not necessarily lead to moral distress. They could even have a protective effect, depending on how the professional faces the situation [61]. Therefore, the individual’s view of a stressful event could determine subsequent psychological complications. For example, someone who believes a case cannot be handled as planned is more likely to be anxious and overwhelmed [64]. A study by Moses *et al*. [60] evaluated moral distress in 889 veterinarians through an online survey in North America, and most participants reported ethical and moral conflict in veterinary medical care in several situations [60]. It was also observed that approximately 70% of the interviewees had little or no preventive training for dealing with conflicting problems, highlighting the need to create tools to mitigate the harmful effects of occupational stressors in veterinary medicine [60].

### Occupational stressors within the sub-specialties

Occupational stressors can also vary according to the field of work [2, 13, 19]. Suicide risk can be increased in those who work in clinical positions, such as surgeons, and small/companion animals’ veterinarians, due to the correlation between work-related stressors and other conditions such as negative effects in undergraduate training, previous life events, genetics, personality dimensions, and psychological morbidity [44].

In the Tomasi *et al*. [19] study, for both male and female veterinarians, the proportionate suicide mortality ratio was higher than expected for those who worked in clinical positions or specialized in companion animals when compared to the general population. Likewise, veterinary surgeons had higher relative mortality ratios than the general population and other healthcare professionals – approximately 4 and 2 times higher, respectively [13]. The same was observed by Nett *et al*. [2], in which veterinarian decedents who specialized in companion, food, and mixed animal medicine were 2.9, 2.0, and 1.7 more likely (respectively) to die by suicide than the general US population.

Despite the aforementioned reports, another study by Dalum *et al*. [44] evaluated 2596 survey responses from Norwegian veterinarians, and no significant correlation between the field of work and serious suicidal thoughts was found, showing the importance of accounting for geographical, cultural, and socioeconomic differences within the occupational risk factors.

### Availability of lethal means for suicide

Environment-related aspects also appear to be important in the occurrence of suicide. It has been reported that people who have easy access to potentially lethal substances and tools, such as firearms, pesticides, and medicine, have higher rates of suicide ideation and/or attempt [2, 65–67]. Clearly, veterinarians are included in this group because many have easy access to anesthetic agents and opioids, and other pharmaceuticals and substances that can easily lead to the conclusion of a suicidal act [68–70]. This is well aligned with results from Witte *et al*. [14], who investigated methods of death for veterinarians’ suicide and found that the most used method was exogenous intoxication (poisoning), followed by the use of a firearm, this second option being the most commonly used by the general population in suicide attempts [14]. In agreement, other studies have pointed to pentobarbital as the most used medication for this purpose, attributable to the availability, and technical knowledge that veterinary professionals possess [14, 68–70]. These studies suggest that strategies to reduce suicide rates should include restricting access to these medications [70, 71], although this could be difficult to implement due to the nature of the job. Yip *et al*. [72] suggested that poor access to lethal methods in a moment of crisis, referred to as “means restriction,” can be a substantial impediment to the act of suicide. This can be a helpful tool in preventive measures for suicide in veterinary professionals considering that many suicides tend to be sudden and unplanned, that is, the individuals tend to use the method most readily accessible to them [72, 73].

It is important to note that possible interventions to reduce modifiable risk factors will require a comprehensive systemic collaboration between policymakers and healthcare professionals to be effective [20]. It does not depend only on individual management but even more on the veterinary community [74]. According to Malvoso [74], key points of suicide prevention in veterinarians include training about tools that bring awareness to suicide risk and incentives for promoting work-life balance. This includes improved organizational skills, promoting a healthy and welcoming work environment, and implementing easily accessible best practice guidelines for suicide risk detection, intervention, and prevention [20].

Furthermore, self-care and social support appear to be fundamental in promoting mental health. Reports from other healthcare-related occupations suggest that self-care is vital in resilience promotion, and a lack of it affects the ability to care for others and can cause professional incompetence [47, 75].

## Suicide Prevention, Resilience, and Mental Health Promotion Strategies

### Recognizing the issue and seeking support

It is necessary to support the use of standard tools for the identification of risk factors and suicide prevention, aiming for the development of emotionally balanced, and socially capable professionals that can offer high-quality services to the community [64, 76, 77].

Increasing awareness on detecting signs of poor mental health and reducing help-seeking stigma are the first measures toward suicide prevention [3, 5]. It is worth mentioning that most poor mental health signs are related to several of the psychological disorders already presented here, and bit is thus essential to seek professional help to identify, diagnose, and properly manage the condition [5, 32].

In the Nett *et al*. [2] study, among the respondents who had current psychological distress, almost 60% reported not receiving treatment for the mental health disorder [2]. However, seeking help depends not only on the individual but also on the people around them, who could aid in identifying signs of a suicidal crisis [74].

### Resilience development

Despite several studies investigating veterinarians’ mental health, little research has been conducted on strategies to improve and promote professional growth and resilience [9, 51]. Some studies have reported that veterinarians are mostly enthusiastic and happy about their work, demonstrating that personal skills and resilience may be directly involved in professional well-being [9, 78, 79].

From the psychological perspective, resilience is complex and multifaceted and refers to the positive process of adapting to adverse and challenging circumstances [51, 80]. It involves an individual’s ability to take advantage of personal and contextual resources to face challenges through a dynamic process. With specific strategies, resilience can lead to positive results, including professional involvement, satisfaction, and well-being [81].

A recent study by Hess-Holden *et al*. [82] involving 4^th^-year veterinary students in the US reported that communication skills could directly influence the development of personal satisfaction. Individuals who communicate more with others about emotional issues, concerns, and tense situations have a greater ability to recognize the suffering of others as well as to be open to empathic concerns compared with individuals with low levels of emotionality [82]. In another study, Cake *et al*. [83] showed that elements of resilience such as emotional competence, social support, motivation, personal resources, organizational culture, and work-life balance support veterinary professionals’ well-being. From the the perspective of veterinary medicine, emotional competence is seen as especially important for exercising resilience, as these professionals face multiple demands, including personal emotional needs; a high processing capacity for these emotions is necessary to successfully use strategies to confront moral distress [83].

Social support, especially from family members, friends, and personal mentors, is critical in the resilience process and is still considered by some authors as the primary precursor of happiness [84, 85]. Such support might be especially paramount in novel professional contexts. Widely described in the literature, moving to a new environment could bring challenges such as lack of support, social isolation, and nostalgia (homesickness), which are considered risk factors for hindered resilience [83].

Motivation is central to resilience because the sense of meaning and purpose provides a personal and contextual resource for reward strategies for stressful situations. Personal resources are defined as “developable systems of positive beliefs about oneself and the world, a feeling of being appreciated and being in control, as well as having skills and attitudes that facilitate these feelings” [86]. Proactivity, self-efficacy, assertiveness, optimism, and reflective behavior are important in the work environment, while autonomy and self-confidence are more important for other environments (e.g., home and social relationships) [83, 86, 87].

### Promoting mental health in the work environment

Dunne *et al*. [88] showed that shared experience of coaching interventions and objective structured clinical examinations have helped empower students to manage assessment-associated anxiety. Like social support, organizational culture has also been shown to be important for workers’ mental health. It depends on supervisors, mentors, and coworkers, autonomy, and opportunities for professional development [89]. A non-judgmental culture, including constant encouragement for seeking help and reduced focus on perfectionism, is also essential for a healthy work environment [83].

Long working hours, high workload, and a disturbance between home and work environments are strongly related to a lack of balance between professional and personal lives [90, 91]. This duality of “lives” should not be compromised for the positivity of one to the detriment of the other since work can and should be a pleasant and rewarding part of life [83].

The occurrence of burnout has frequently been recognized when discussing stress at the workplace. Positive improvements in working conditions, such as reduced workload, healthy interpersonal relationships, appreciation of professionals, avoiding monotony, and improvements in social and physical conditions in the occupational environment, demonstrate good results in preventing burnout [77, 92]. Preventive measures should be used to avoid situations such as abandonment, low-quality work, interpersonal conflicts, and their consequences for workers, customers, and institutions [77]. In this case, prevention and coping strategies for the individual should focus on the exercise of self-care (physical, emotional, or mental health). Such strategies require acknowledging the problem and its evaluation to facilitate learning effective coping strategies during stressful situations [8, 93], and allowing for compassion. Some authors indicate that compassion is a skill that can be refined, starting with transforming empathy as a way to reverse empathic suffering into a positive feeling [94]. To gradually refine compassion, meditation exercises and participation in interactive therapy groups are indicated [94]. They can make the individual less focused on negative feelings and bring the ability to continue feeling empathy for the suffering of others without turning it against oneself [95]. Several studies have shown that compassion training has lasting effects, and it facilitates the transposition of prosocial attitudes and behavior to other people and situations [96, 97].

Diagnosis, intervention, and prevention programs related to the work environment should be based on three levels: individual response, occupational context, and the interaction between them. From a personal perspective, the approach consists of learning adaptive coping strategies in stressful situations, mainly to prevent adverse effects. In the occupational context, the issues related to the organization should be addressed to improve the work environment. Finally, when combined, the approach should be bidirectional and integrated [93].

According to the Future Leaders Program of the AVMA [98], “an intentional re-shaping of workplace culture must incorporate active communication with an effort to reduce conflict and promote unity.” To improve veterinarians’ satisfaction and well-being in the work environment, it is necessary to consider three dimensions: new hire training, performance feedback, and team meeting [98].

The training of newly hired employees is important to develop connections, team relationships, acquisition of knowledge, and technical skills for work. In addition, providing valuable assessment to employees is essential, and it should be applied by incorporating tools that include best practices for communication and formal feedback session recommendations. By implementing a feedback system that integrates the most effective communication and self-awareness practices, veterinary professionals can foster stability and promote positive growth in themselves and their teams [98].

Effective team meetings can yield great results, but they demand time and effort. A veterinary team can conquer complicated challenges and generate effective solutions when equipped with strong communication skills, motivation, and collaboration. Competitions can also be an enjoyable way to break from routine business tasks during team meetings and to promote bonding and trust-building among colleagues while uncovering their strengths. Numerous team challenges can encourage creativity, cooperation, and communication while enhancing problem-solving abilities. When organizing teams for these activities, it is recommended that diversity be fostered by including veterinarians from different sub-specialties, including technicians, assistants, and receptionists in each team, when possible [98].

Welfare and suicide prevention programs are currently available to the veterinary community in many countries. They are important because they significantly impact the suicide rate, as suggested in a study by Knox *et al*. [99]. Thus, academic and professional institutions should include integrated and holistic approaches to physical, emotional, and mental well-being to facilitate the development of personal skills to handle the stress of veterinary students and professionals [74, 85, 86, 100].

## Conclusion

Veterinarians are frequently exposed to stressful situations and other occupational challenges, placing them at a high risk of developing mental health disorders. It is known that depression is the major risk factor associated with suicide ideation and suicide attempts; therefore, it is crucial to determine predisposing and triggering factors to this psychological disorder to establish preventive measures. Significant occupational risks in veterinary medicine include high debt, poor work-life balance, and decision-making around/performing euthanasia. More studies are needed to understand the risk factors for suicide in veterinary professionals. For example, understanding the relationship between socioeconomic and cultural influences on the mental health of veterinarians is lacking in the literature.

Future work on practical tools for preventing poor mental health and suicide in veterinary medicine is urgently needed. Social support, personal and social skills training, and compassion are thought to be effective precursors of resilience. Due to the high suicide rate among veterinarians, preventive and training programs should be incorporated in universities and other institutions to promote well-being and prevent psychological disorders and suicide, especially early in the professional’s career. In addition, improving work culture is important, focusing on compassionate and non-judgmental assessment of employees, providing training, effective feedback and team meetings or activities to encourage diversity, creativity, cooperation, and communication within the work environment. The emphasis should not be limited to the self-recognition of mental health disorders but should be on seeking early specialized psychological care. Actions to promote well-being should be routine, starting during veterinarian’s training.

## Authors’ Contributions

CRS: Conducted the literature review and drafted the manuscript. AADG, TRSD, RFCV, ACA, and ARSS: Drafted the manuscript and critically revised it for important intellectual content. All authors have read, reviewed, and approved the final manuscript.
